# Association of Birth Order With Mental Health Problems, Self-Esteem, Resilience, and Happiness Among Children: Results From A-CHILD Study

**DOI:** 10.3389/fpsyt.2021.638088

**Published:** 2021-04-14

**Authors:** Yoshifumi Fukuya, Takeo Fujiwara, Aya Isumi, Satomi Doi, Manami Ochi

**Affiliations:** ^1^Department of Global Health Promotion, Tokyo Medical and Dental University, Tokyo, Japan; ^2^Department of Health and Welfare Services, National Institute of Public Health, Saitama, Japan

**Keywords:** birth order, mental health, resilience, happiness, self-esteem

## Abstract

**Objective:** This study aimed to investigate the association of birth order with mental health problems, self-esteem, resilience, and happiness among children aged 9–10 years.

**Methods:** This was a cross-sectional study using data from the Adachi Child Health Impact of Living Difficulty (A-CHILD) study, which was a population-based study of children in fourth grade in public schools in Adachi City, Tokyo, Japan in 2018 (*N* = 3,744). Parent-rated Strengths and Difficulties Questionnaire (SDQ) and self-rated resilience, happiness, and self-esteem score were used to assess child mental health. Multiple regression analysis for continuous outcomes and logistic regression for dichotomous outcomes were used to examine the association of birth order with mental health problems, resilience, happiness, and self-esteem. The analysis was controlled for child sex, mother's age, mother's education, caregiver's depressive symptoms, household income, and living with grandparents.

**Results:** Last-borns showed the lowest total difficulties score in the SDQ, while only children showed the highest (*p* < 0.001). The highest prosocial behaviors score was found among last-borns (*p* < 0.001). Resilience score was also highest among last-borns, followed by first-borns, middle-borns, and only children. The lowest happiness score was found among middle-borns. Self-esteem score did not differ by sibling types. These associations were similar in the adjusted model and dichotomized outcomes model.

**Conclusions:** Differential impacts of birth order on child mental health, for both positive and negative sides, were found. Further research is warranted to elucidate the mechanism of the association between birth order and the development of behavior problems and the positive aspects such as resilience, happiness, and self-esteem among children.

## Introduction

There is an increasing number of mental health problems among adolescents, with 10–20% of them estimated to suffer from these problems in the world (1). In addition, half of the cases of lifetime mental health problems begin by the age of 14 (2). Japan is not exception; for example, the number of suicide among children and adolescents has gradually increased over a decade (3), ranked second in Asia, followed by Korea (4). To reveal this situation, it is crucial to understand risk and protective factors for prevention of mental illness in adolescence and adulthood.

Previous research has shown that later-born children aged 7–12 years in the US have been found to have a higher level of depression and anxiety (5). A population-based study in the UK has shown that later-born adolescents are more likely to have suicide attempts and psychiatric problems (6). Similarly, prior register-based studies have reported that later-born adults aged 16 years are at a higher risk of suicidal behavior in Norway and Sweden (7, 8). These findings from Western countries have indicated later-born have an increased risk of mental health problems. There are several considerations underling the mechanisms of the associations. These include later-born children being more likely to have limited interactions with parents, less parental attention, and diluted available home resources (9, 10). These circumstances have adverse effects on mental health throughout sensitive development periods in childhood, which might last across the life course. To date, however, few epidemiological studies have examined whether birth order is associated with mental health problems among children, particularly, pre-adolescent children. Further, most of the previous studies were conducted in Western countries. There is a lack of empirical studies to investigate the associations in non-Western countries, including Japan.

Positive mental health refers to a state of subjective well-being and functioning well (11). Resilience (12) and self-esteem (13) can be considered important aspects of positive mental health (14–16). Further, happiness is known to be one of the indicators based on an individual's hedonistic view or emotional well-being, which suggests positive affectivity and satisfaction with life (17). Previous studies in the US have indicated that first-born adolescents are more likely to have higher self-esteem than other siblings and only children (18), whereas middle-born adolescent males are found to have lower self-esteem (19). Moreover, other research has reported that last-born and only children are happier than first-and middle-born among US young children (20). However, it is still unknown whether these findings in the US enable to be generalized to any other population with different sociocultural characteristics, such as Japan. Indeed, the association of birth order with positive aspects in non-Western countries remains unknown due to the lack of research. Furthermore, limited population-based studies focusing on children have been conducted to examine the association.

Japan has unique sociocultural characteristics affecting child-rearing. Japan had the i.e., system prescribed a patriarchal and primogeniture system in place, which was abolished in 1947. Nonetheless, the i.e., system still influences family relationships to some extent (21, 22). In the familial contexts, Japanese parents may assign a perceived ideal role to their children within a family according to the birth order; in particular, the parents require the older siblings to behave more maturely and responsibly, including taking care of younger siblings (23). Second, Japan has a specific concept of parent-child relationships, known as *amae* (24). *Amae* refers to the feeling of dependence on others and the presumption on others' acceptance and indulgence (24, 25), which is somewhat similar to the secure base of attachment in terms of needs for closeness and security (22, 26, 27). *Amae* has influenced Japanese values, which makes a difference in child-rearing practice, compared to other countries. For example, Japanese parents tend to indulge their children and have closer physical proximity to them (23, 28), whereas parents in Western countries are likely to encourage their children to be independent and behave autonomously from an early age (29–31). Furthermore, according to the i.e., system and *amae*, Japanese parents may modify their child-rearing practice according to birth order (32, 33). Indeed, first-borns are more likely to be subjected to stricter upbringing, whereas last-born is to be well-taken care of by their parents and older siblings (32–34). Given the differences in parenting practice between siblings, the unique sociocultural characteristics may influence the association of birth order with child development and mental health. A prior study among pre-school children in Japan reported that birth order was associated with developing children's self-reliance (34). To date, however, there is still uncertainty whether birth order has effects on mental health among Japanese pre-adolescent, say, aged 9–10 years old.

Over the years, family structures have changed in developed countries, including Japan. For example, birth rates have decreased (35). That is, more children have fewer siblings or none. Thus, data reflecting current family contexts is needed to assess the association between birth order and mental health among children. However, few empirical studies have investigated the association in recent years. This study aimed to examine the associations of birth order with mental health problems, and happiness, self-esteem, and resilience among children aged 9–10 years in Japan, using a population-based dataset collected in 2018.

## Materials and Methods

### Participants

**Figure 1:**
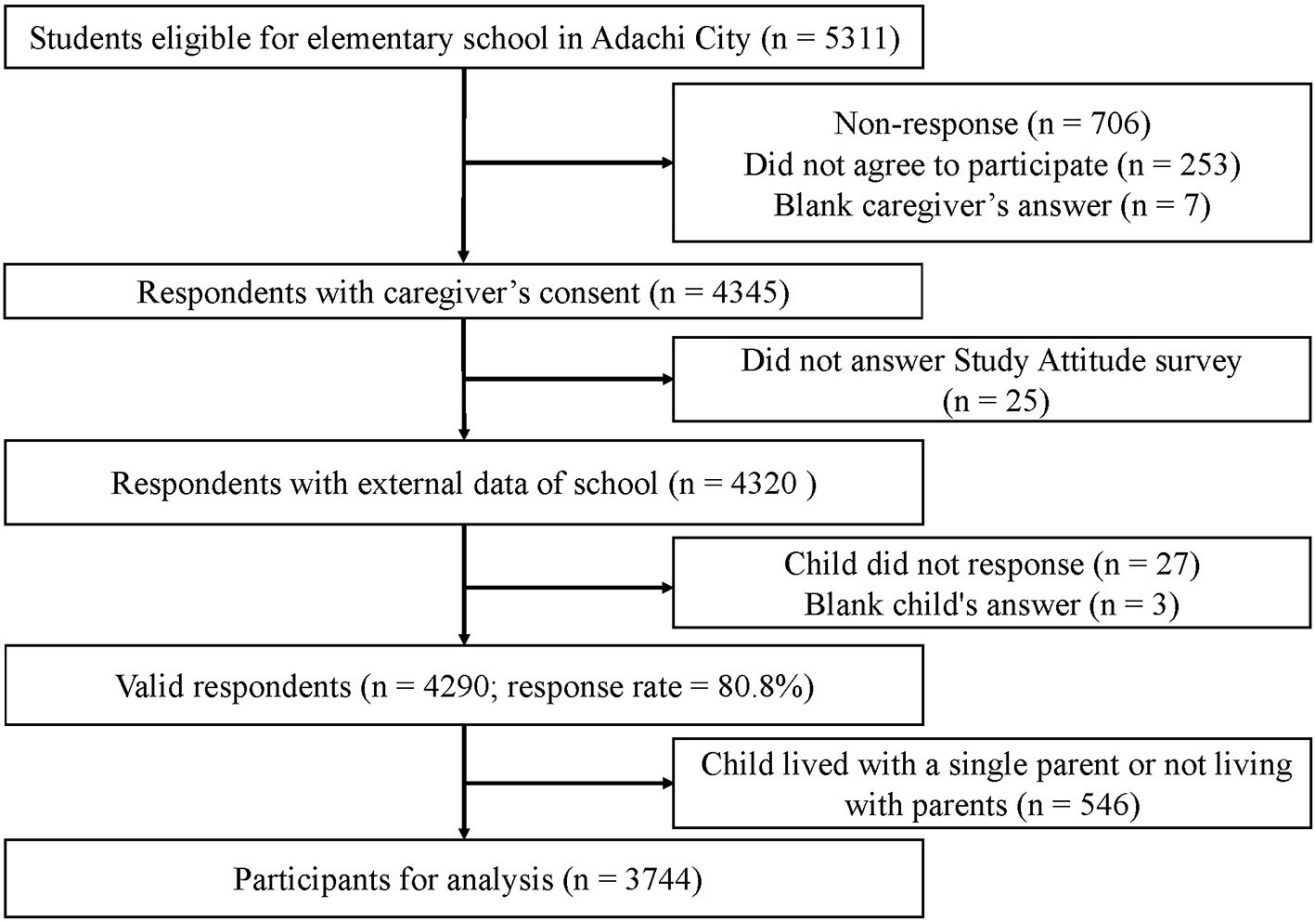
Flowchart of participants.

We used data from the Adachi Child Health Impact of Living Difficulty (A-CHILD) study, conducted in all 69 public elementary schools in collaboration with city hall and educational committee in Adachi City, Tokyo, Japan (36). This survey was a longitudinal complete-sample survey started in 2015 as a first-wave for all first-grade children in the schools. In 2018 as a third-wave, questionnaires were distributed to all children aged 9–10 years (fourth grade) attending the schools (*n* = 5,311). Children responded to the self-reported questionnaire at school and took the questionnaire home so that their caregivers answered it. The questionnaire completed by the caregivers was submitted to the school anonymously. A total of 4,290 participants were eligible (response rate = 80.8%). We excluded children who lived with a single parent or not living with parents (*n* = 546, 12.7% of respondents). Finally, a sample of, 3,744 children and caregivers were used for analysis (Figure 1).

### Explanatory Variable

#### Birth Order

The caregivers provided information regarding the children's birth order. Based on previous birth order studies (37, 38), each child was classified into four categories based on individual's birth order: only child (no siblings), first-born (having only younger siblings), middle-born (having both older and younger siblings), and last-born (having only older siblings).

### Outcome Variables

#### Mental Health

Child mental health problems were assessed by the caregivers using the Japanese version of the Strengths and Difficulties Questionnaire (SDQ) (39). The reliability and validity of the SDQ in Japanese children were documented (40). The SDQ includes five items, emotional symptoms, conduct problems, hyperactive/inattention, peer relationship problems, and prosocial behaviors. The total difficulties score was calculated by the sum of the four items except for prosocial behavior, which was used as a continuous outcome. Each item has cut-off scores for normal, borderline, and clinical range; 0–12, 13–15, and 16–40 for total difficulties score; 0–3, 4, and 5–10 for emotional symptoms score; 0–3, 4, and 5–10 for conduct problems score; 0–5, 6, and 7–10 for hyperactivity/inattention score; 0–3, 4, and 5–10 for peer problems score; and 6–10, 5, and 0–4 for prosocial behavior score (41). The score of each item was dichotomized to use as a binary variable (normal and borderline range = 0 and clinical range = 1) for statistical analysis.

#### Self-Esteem

Children were asked about their self-esteem using the subscales from the Japanese version of the Children's Perceived Competence Scale (CPCS) (42), based on the Perceived Competence Scale for Children (43). The subscales consist of 10 items, for example, “are you satisfied with the way you are now?” or “do you think you have few good points?”. The Cronbach's alpha for the scale was 0.84 in this study. Each item was rated from 1 (no), 2 (rather no), 3 (rather yes), and 4 (yes). Each score was summed and used as a continuous outcome. A higher total score indicated a higher level of self-esteem. The community-based survey reported that the percentage of low self-esteem regarding eight questions related to self-esteem was averagely 7.2% among children aged 10–11years and 8.3% among those aged 13–14 years (44). Further, according to a national survey of Ministry of Education, Culture, Sports, Science and Technology, the percentage of low self-esteem regarding one question related to self-esteem was 6.4% among primary school children and 8.4% among junior high school children on average over 6 years from 2014 to 2019 (45). These results suggest the percentage of children with low self-esteem could be 10% or less. In this study, the percentage of the score < the 10th percentile was 8.4%. Referring to these findings, we defined the score < the 10th percentile as low self-esteem because the 10th percentile cut-off level could incorporate children with low self-esteem.

#### Resilience

The Children's Resilient Coping Scale (CRCS) was used to assess child resilience (46). This scale consists of eight items, for example, “speaks positively about their future,” “able to get ready for school, study,” and “do his/her chores without directions,” or “able to give up on things they want or do things that they do not like to do for better future outcomes.” The scale has high internal consistency (46). The caregivers rated their child resilience from zero (never) to four (very frequently). The Cronbach's alpha for the scale was 0.84 in this study. Each score of the eight items was summed, ranging from 0 to 32, and used as a continuous outcome. A higher total score indicated higher resilience. The distribution was left-skewed (more children rating higher scores ≥ in the 10th percentile). Then, the total score of resilience was divided into two categories below the 10th percentile as low resilience to capture a very low level of resilience.

#### Happiness

Children were asked about their happiness by the question, “how happy do you think you are?” which was rated from 0 (not happy) to 10 (very happy) and used as a continuous outcome. Due to the left-skewed distribution, more children rated higher scores ≥ in the 10th percentile. Indeed, the proportion < the 10th percentile was 5.6%. Further, a community-based survey conducted in Tokyo in 2016 showed that 5.1% of adolescents perceived low happiness (44), which was similar to our proportion of the score < the 10th percentile. Referring to the findings, we defined the score < the 10th percentile as low happiness.

### Other Variables

We selected potential confounding variables based on previous research investigating birth order and mental health problems (6–8, 47). The following variables were included in this study: child's sex (boys or girls), household income (<3.0, 3.0 ≤ 6.0, 6.0 ≤ 10.0, 10.0+ million JPY and unknown; 110 JPY≈ 1 USD), mother's age (Seventy-eight missing data were imputed with the mean of the age) and education attainment (high school graduate or less, some college, college or university graduate, others/unknown), parental history of psychiatric disorders (yes or no), respondent's psychological distress, living with grandparents, and children's school absence due to illness and trauma. The respondent's psychological distress was assessed using the Japanese version of the Kessler 6 (K6), which consisted of six items with a 5-point Likert scale (48). The sum of each score was calculated (range: 0–24); a score of 5–12 was defined as moderate psychological distress, and a score of ≥13 was defined as severe. As for living with grandparents, prior studies have indicated that grandparents' support is related to women's fertility decisions (49, 50). Also, grandparents raising are found to affect grandchildren's mental health (51–53). Thus, living with grandparents was included. Also, previous research has shown the association of birth order with PTSD (54) and illness such as metabolic problems (55), allergic symptoms (56), and respiratory diseases (57), which are known to relate to children's mental health (58–60). According to these findings, we included children's school absence due to illness and trauma in this analysis. Regarding parental age and education, the mother's age and education attainment correlated with the father's ones (*r* = 0.40 and 0.65, respectively). Further, in Japan, mothers have more child care time than fathers and play the primary role in child development in daily life (61). Accordingly, we did not take father's age and education attainment into consideration.

### Statistical Analysis

First, we compared the characteristics among the four groups: only child, first-borns, middle-borns, and last-borns. Second, we treated the outcomes as continuous variables; that is, we calculated the mean of the scores of the SDQ, self-esteem, happiness, and resilience, and compared by analysis of variance (ANOVA) with *post-hoc* pairwise comparison using Bonferroni's correction. Third, multiple linear regression analyses were used to examine the association between birth order and mental health problems using each SDQ subscales score, the total difficulties score, the self-esteem score, the happiness score, and the resilience score. The only child was treated as a reference group category. In the adjusted model, covariates (children's sex, mother's age, mother's education, parental history of psychiatric disorders, respondent's psychological distress, household income, living with grandparents, and children's school absence due to illness and trauma) were included. Further, to investigate the association between birth order and the clinical or severe range of the outcomes, multiple logistic regression analyses were performed. These covariates were added in the adjusted model, and we confirmed that multicollinearity is unlikely (all variance inflation factor <2.0). Missing data were substituted by dummy variables. A *p*-value <0.05 was considered as the level of statistical significance. We used STATA version 15.0 (StataCorp., College Station, TX, USA) for all analyses and followed the Strengthening the Reporting of Observational Studies in Epidemiology Statement (STROBE) guidelines.

### Ethics Statement

This study was approved by the Ethics Committee at the National Center for Child Health and Development (Study ID: 1147) and Tokyo Medical and Dental University (Study ID: M2016-284).

## Results

### Demographics

Table 1 shows the characteristics of the participants. There were 1,278 first-borns (34.1%), 466 middle-borns (12.5%), 1,356 last-borns (36.2%), and 644 only children (17.2%). Almost half of the children were boys (*n* = 1,908, 51.0%). With regard to mother's age and educational attainment, most of the mothers were 40–44 years old and graduated some college. As for household income, the largest group was between 6 and 10 million JPY. As for caregiver's mental health, the mean scores of K6 showed no difference across all groups. Regardless of the birth order, more than 90% of children lived without grandparents. Approximately 7% of caregivers reported to have psychiatric disease history, and 34% of children had experienced to be absent from school due to illness or trauma.

**Table 1:** Demographic characteristics.

	**Overall**		**Only child**		**First-born**		**Middle**		**Last-born**	
	**(*****n*** **= 3,744)**		**(*****n*** **= 644, 17.2%)**		**(*****n*** **= 1,278, 34.1%)**		**(*****n*** **= 466, 12.5%)**		**(*****n*** **= 1,356, 36.2%)**	
	***n***	**%**	***n***	**%**	***n***	**%**	***n***	**%**	***n***	**%**
**Sex**										
Boys	1,908	51.0	324	50.3	694	54.3	234	50.2	656	48.4
Girls	1,836	49.0	320	49.7	584	45.7	232	49.8	700	51.6
**Mother's educational attainment**										
High school graduate or less	956	25.5	175	27.2	284	22.2	137	29.4	360	26.6
Some college	1,248	33.3	235	36.5	427	33.4	136	29.2	450	33.2
College or University graduate	610	16.3	94	14.6	265	20.7	58	12.5	193	14.2
Others/unknown	930	24.8	140	21.7	302	23.6	135	29.0	353	26.0
**Mother's age** (Mean, SD)	41.2	4.9	43.1	4.7	38.9	4.5	39.6	4.5	43.1	4.3
**Respondent's mental health (K6)**										
<5	2,480	66.2	426	66.2	835	65.3	308	66.1	911	67.2
5 ≤ 13	989	26.4	175	27.2	344	26.9	115	24.7	355	26.2
13+	162	4.3	32	5.0	56	4.4	25	5.4	49	3.6
Missing	113	3.0	11	1.7	43	3.4	18	3.9	41	3.0
**Parental history of psychiatric disorder**										
No	3,472	92.7	574	89.1	1,185	92.7	430	92.3	1,283	94.6
Yes	272	7.3	70	10.9	93	7.3	36	7.7	73	5.4
**Household income (million yen)**										
<3.0	187	5.0	33	5.1	62	4.9	30	6.4	62	4.6
3.0 ≤ 6.0	1,181	31.5	211	32.8	411	32.2	163	35.0	396	29.2
6.0 ≤ 10.0	1,382	36.9	238	37.0	477	37.3	153	32.8	514	37.9
10.0+	484	12.9	94	14.6	160	12.5	43	9.2	187	13.8
Missing	510	13.6	68	10.6	168	13.2	77	16.5	197	14.5
**Living with grandparents**										
No	3,432	91.7	597	92.7	1,182	92.5	422	90.6	1,231	90.8
Yes	312	8.3	47	7.3	96	7.5	44	9.4	125	9.2
**School absences due to illness or trauma**										
No	2,448	65.4	429	66.6	796	62.3	306	65.7	917	67.6
Yes	1,296	34.7	215	33.4	482	37.7	160	34.3	439	32.4
**Child mental health**										
**SDQ**										
**Total difficulties**										
Normal	2,715	72.5	450	69.9	892	69.8	346	74.3	1,027	75.7
Borderline	411	11.0	79	12.3	151	11.8	45	9.7	136	10.0
Clinical	495	13.2	102	15.8	191	15.0	56	12.0	146	10.8
Missing	123	3.3	13	2.0	44	3.4	19	4.1	47	3.5
**Emotional symptoms**										
Normal	2,954	78.9	499	77.5	996	77.9	383	82.2	1,076	79.4
Borderline	302	8.1	61	9.5	104	8.1	27	5.8	110	8.1
Clinical	365	9.8	71	11.0	134	10.5	37	7.9	123	9.1
Missing	123	3.3	13	2.0	44	3.4	19	4.1	47	3.5
**Conduct problems**										
Normal	2,860	76.4	509	79.0	938	73.4	348	74.7	1,065	78.5
Borderline	335	9.0	60	9.3	130	10.2	39	8.4	106	7.8
Clinical	426	11.4	62	9.6	166	13.0	60	12.9	138	10.2
Missing	123	3.3	13	2.0	44	3.4	19	4.1	47	3.5
**Hyperactivity/inattention problems**										
Normal	2,997	80.1	511	79.4	998	78.1	375	80.5	1,113	82.1
Borderline	237	6.3	43	6.7	95	7.4	20	4.3	79	5.8
Clinical	388	10.4	77	12.0	141	11.0	53	11.4	117	8.6
Missing	122	3.3	13	2.0	44	3.4	18	3.9	47	3.5
**Peer relationship problems**										
Normal	2,996	80.0	464	72.1	1,024	80.1	384	82.4	1,124	82.9
Borderline	285	7.6	80	12.4	90	7.0	25	5.4	90	6.6
Clinical	340	9.1	87	13.5	120	9.4	38	8.2	95	7.0
Missing	123	3.3	13	2.0	44	3.4	19	4.1	47	3.5
**Prosocial behavior**										
Normal	2,596	69.3	452	70.2	850	66.5	320	68.7	974	71.8
Borderline	533	14.2	89	13.8	187	14.6	72	15.5	185	13.6
Clinical	492	13.1	90	14.0	197	15.4	55	11.8	150	11.1
Missing	123	3.3	13	2.0	44	3.4	19	4.1	47	3.5
**Self-esteem score**
Not low	3,299	88.1	570	88.5	1,118	87.5	415	89.1	1,196	88.2
Low	316	8.4	54	8.4	111	8.7	35	7.5	116	8.6
Missing	129	3.5	20	3.1	49	3.8	16	3.4	44	3.2
**Resilience score**
Not low	3,457	92.3	583	90.5	1,183	92.6	427	91.6	1,264	93.2
Low	285	7.6	61	9.5	95	7.4	38	8.2	91	6.7
Missing	2	0.1	0	0.0	0	0.0	1	0.2	1	0.1
**Happiness score**
Not low	3,376	90.2	589	91.5	1,138	89.1	407	87.3	1,242	91.6
Low	208	5.6	30	4.7	78	6.1	38	8.2	62	4.6
Missing	160	4.3	25	3.9	62	4.9	21	4.5	52	3.8

### Outcome Variables by Birth Order

Table 2 presents the mean scores of the SDQ, self-esteem, happiness, and resilience for birth order. Last-borns showed lower score on total difficulties, conduct problems and hyperactivity/inattention (*p* < 0.001, <0.001, and <0.002, respectively) and higher score on prosocial behavior and resilience, compared to other groups (*p* < 0.001 and <0.001). First-borns showed a higher score of conduct problems than other groups (*p* < 0.001). The happiness score of middle-borns was the lowest in all groups (*p* < 0.002). There were no differences in the self-esteem scores among the four groups.

**Table 2:** Results of the ANOVA between birth order and mental health problems self-esteem, resilience and happiness.

	**Overall**	**Only child**	**First-born**	**Middle**	**Last-born**	***F***	***p*-value**	**ω^2^**	***p*****-value for** ***post-hoc*** **pairwise comparison**					
	**Mean** **(SD)**	**Mean** **(SD)**	**Mean** **(SD)**	**Mean** **(SD)**	**Mean** **(SD)**				**O vs. F**	**O vs. M**	**O vs. L**	**F vs. M**	**F vs. L**	**M vs. L**
**SDQs**														
Total difficulties score	9.26 (5.45)	9.98 (5.56)	9.58 (5.59)	8.92 (5.58)	8.74 (5.15)	9.70	<0.001	0.007	0.818	0.010	<0.001	0.162	0.001	1.000
Emotional symptoms score	1.87 (1.89)	2.00 (1.91)	1.93 (1.95)	1.73 (1.86)	1.81 (1.83)	2.77	0.04	0.002	1.000	0.117	0.213	0.297	0.582	1.000
Conduct problems score	2.22 (1.80)	2.16 (1.80)	2.36 (1.83)	2.29 (1.82)	2.08 (1.75)	6.63	<0.001	0.005	0.058	1.000	1.000	1.000	<0.001	0.185
Hyperactivity/inattention score	3.26 (2.33)	3.43 (2.37)	3.38 (2.37)	3.23 (2.40)	3.08 (2.23)	4.82	0.002	0.003	1.000	1.000	0.013	1.000	0.007	1.000
Peer relationship problems score	1.90 (1.77)	2.39 (1.91)	1.88 (1.81)	1.67 (1.67)	1.77 (1.63)	21.44	<0.001	0.017	<0.001	<0.001	<0.001	0.192	0.637	1.000
Prosocial behavior score	6.71 (2.09)	6.54 (2.09)	6.56 (2.12)	6.81 (2.16)	6.89 (2.03)	7.02	<0.001	0.005	1.000	0.203	0.003	0.179	0.001	1.000
Self-esteem score	18.1 (6.07)	18.2 (6.15)	18.3 (6.17)	17.7 (5.85)	17.9 (6.02)	1.54	0.20	0.0004	1.000	0.748	1.000	0.363	0.967	1.000
Resilience score	22.20 (5.23)	21.55 (5.29)	22.23 (5.26)	21.96 (5.33)	22.55 (5.10)	5.73	<0.001	0.004	0.039	1.000	<0.001	1.000	0.724	0.218
Happiness score	8.16 (2.11)	8.29 (2.04)	8.20 (2.12)	7.81 (2.34)	8.18 (2.04)	4.97	0.002	0.003	1.000	0.002	1.000	0.006	1.000	0.010

*O, Only child; F, First-born; M, Middle; L, Last-born. Statistical significance: p-value < 0.05*.

**Figure 2:**
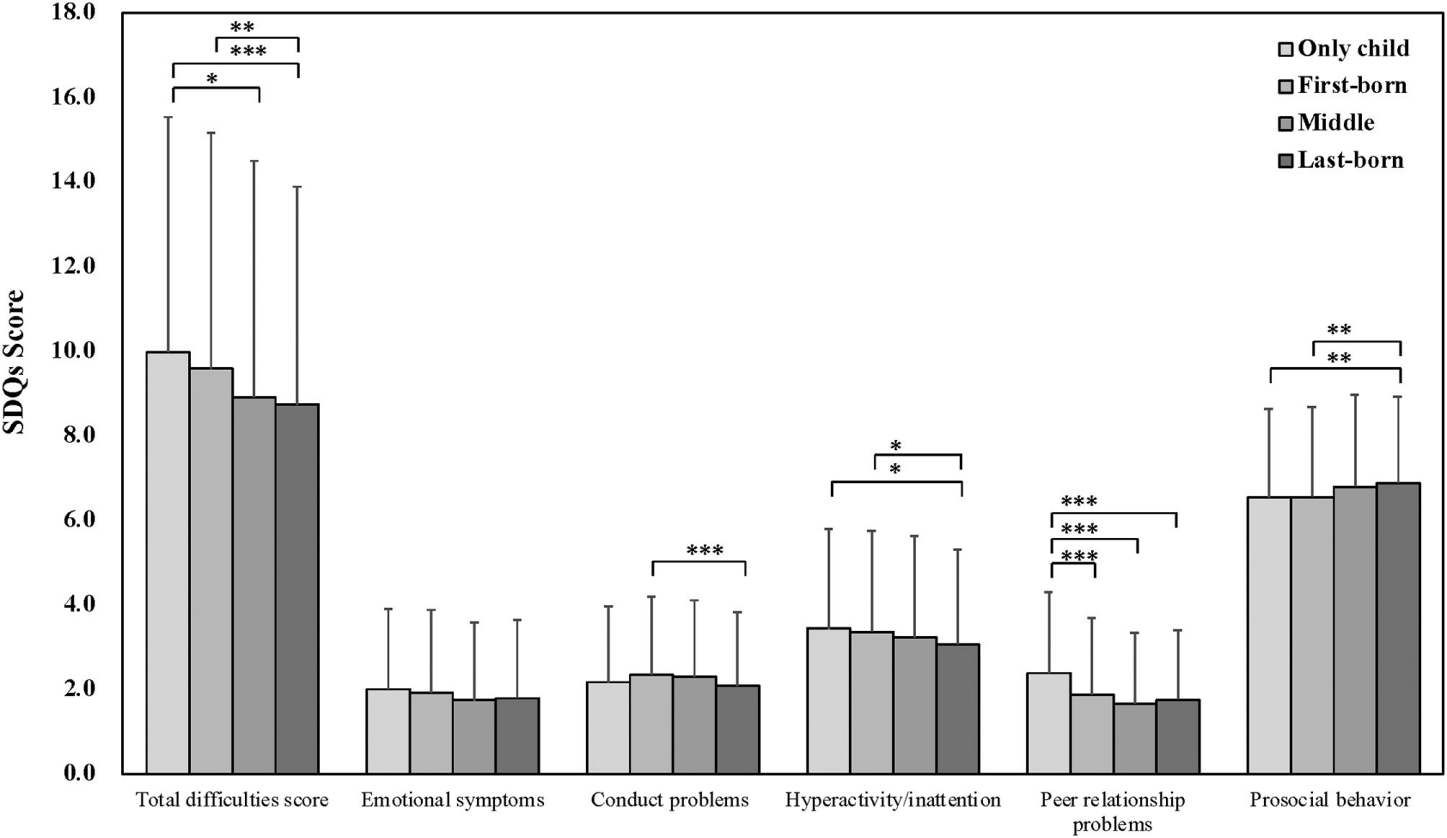
Association of birth order and the SDQ by ANOVA with the *post-hoc* pairwise comparison. ***p < 0.001; **p < 0.01; *p < 0.05.

**Figure 3:**
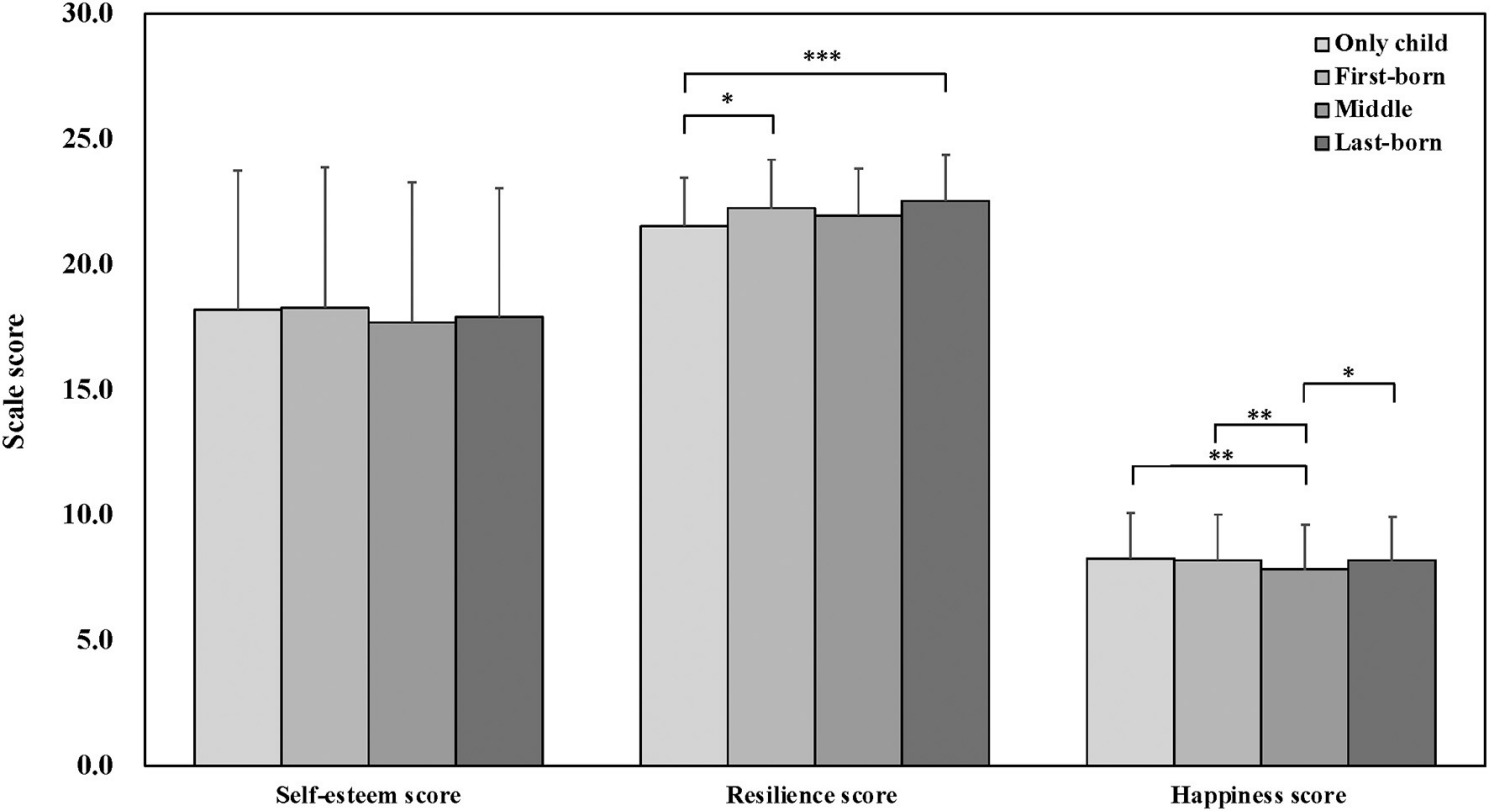
Association of birth order and Self-esteem, Resilience, and Happiness by ANOVA with the *post-hoc* pairwise comparison. ***p < 0.001; **p < 0.01; *p < 0.05.

Further, the results of the *post-hoc* pairwise comparison showed *p*-value for the association between categories of birth order (Figures 2, 3). As for the SDQ, a significant difference was observed in the total difficulties score between only children and middle-borns, and only children and last-borns; hyperactivity/inattention between only children and last-borns: peer relationship problems between only children and each categories; prosocial behavior between only children and last-borns. As for the self-esteem score, there were no difference between only children and each categories. Moreover, a significant difference was observed in the resilience score between only children and first-borns, and only children and last-borns; the happiness score between only children and middle-borns.

### Regression Analyses

Table 3 shows multivariate linear regression and logistic regression analysis examining the association of birth order with scores of the SDQ, self-esteem, happiness, and resilience. As for linear regression analysis, the score of total difficulties and peer relationship problems in last-borns, middle-borns, and first-borns were significantly lower than only children as the reference group in the adjusted model. Table 3 also presents the significant association between last-borns and lower score of hyperactivity/inattention and higher score of prosocial behavior (β = −0.06, *p* < 0.01; β = 0.07, *p* < 0.01), middle-borns and lower score of emotional symptoms and happiness (β = −0.06, *p* < 0.01; β = −0.08, *p* < 0.001), and first-borns and higher score of conduct problems (β = 0.05, *p* = 0.03). The resilience score is significantly higher in last-borns (β = 0.08, *p* < 0.001) and first-borns (β = 0.05, *p* = 0.03). The self-esteem score was not associated with birth order.

**Table 3:** Associations between birth order and mental health problems, self-esteem, resilience, and happiness in linear and logistic regression.

	**First-born**							**Middle**							**Last-born**							***R*^2^^a^**	**Power^a^**	***F*^a^**
	**Linear regression**				**Logistic regression**			**Linear regression**				**Logistic regression**			**Linear regression**			**Logistic regression**						
	***B*** **(SE)**	**β**	***t***	***p***	***B*** **(SE)**	**OR** **(95%CI)**	***p***	***B*** **(SE)**	**β**	***t***	***p***	***B*** **(SE)**	**OR** **(95%CI)**	***p***	***B*** **(SE)**	**β**	***t***	***p***	***B*** **(SE)**	**OR** **(95%CI)**	***p***			
**SDQ**																								
**Total difficulties score**																								
Unadjusted	−0.40 (0.27)	−0.03	−1.49	0.136	−0.05 (0.13)	0.95 (0.73–1.23)	0.700	−1.06 (0.34)	−0.06	−3.15	0.002	−0.30 (0.18)	0.74 (0.52–1.06)	0.097	−1.24 (0.26)	−0.11	−4.70	<0.001	−0.43 (0.14)	0.65 (0.50–0.86)	0.002	0.008	1.00	9.70^***^
Adjusted^b^^,^^d^	−0.52 (0.26)	−0.05	−1.99	0.047	−0.11 (0.15)	0.90 (0.67–1.20)	0.469	−1.25 (0.32)	−0.08	−3.89	<0.001	−0.42 (0.19)	0.66 (0.45–0.95)	0.027	−1.09 (0.25)	−0.10	−4.38	<0.001	−0.40 (0.15)	0.67 (0.50–0.89)	0.005	0.130	1.00	30.34^***^
**Emotional symptoms score**																								
Unadjusted	−0.07 (0.09)	−0.02	−0.74	0.462	−0.04 (0.16)	0.96 (0.71–1.30)	0.797	−0.27 (0.12)	−0.05	−2.34	0.019	−0.34 (0.21)	0.71 (0.47–1.08)	0.110	−0.19 (0.09)	−0.05	−2.10	0.036	−0.20 (0.16)	0.82 (0.60–1.11)	0.202	0.002	0.823	2.77^*^
Adjusted^b^^,^^d^	−0.11 (0.09)	−0.03	−1.21	0.226	−0.16 (0.17)	0.85 (0.61–1.19)	0.349	−0.34 (0.11)	−0.06	−3.01	0.003	−0.52 (0.22)	0.60 (0.38–0.93)	0.021	−0.15 (0.09)	−0.04	−1.76	0.078	−0.17 (0.16)	0.85 (0.61–1.17)	0.305	0.096	1.00	21.22^***^
**Conduct problems score**																								
Unadjusted	0.23 (0.09)	0.06	2.59	0.010	0.36 (0.16)	1.43 (1.05–1.94)	0.024	0.13 (0.11)	0.02	1.21	0.225	0.35 (0.19)	1.42 (0.98–2.08)	0.067	−0.08 (0.09)	−0.02	−0.89	0.372	0.08 (0.16)	1.08 (0.79–1.48)	0.627	0.006	0.994	6.63^***^
Adjusted^b^^,^^d^	0.20 (0.09)	0.05	2.18	0.030	0.27 (0.17)	1.31 (0.94–1.83)	0.106	0.09 (0.11)	0.02	0.84	0.401	0.27 (0.20)	1.31 (0.88–1.94)	0.181	−0.05 (0.09)	−0.01	−0.58	0.563	0.14 (0.16)	1.14 (0.83–1.58)	0.412	0.058	1.00	12.33^***^
**Hyperactivity/inattention score**																								
Unadjusted	−0.05 (0.11)	−0.01	−0.41	0.684	−0.07 (0.15)	0.93 (0.69–1.25)	0.621	−0.19 (0.14)	−0.03	−1.35	0.177	−0.04 (0.19)	0.97 (0.66–1.40)	0.853	−0.35 (0.11)	−0.07	−3.08	0.002	−0.35 (0.16)	0.71 (0.52–0.96)	0.025	0.004	0.968	4.82^**^
Adjusted^b^^,^^d^	−0.11 (0.11)	−0.02	−0.96	0.339	−0.17 (0.16)	0.84 (0.61–1.16)	0.298	−0.27 (0.14)	−0.04	−1.92	0.055	−0.13 (0.20)	0.88 (0.59–1.30)	0.522	−0.29 (0.11)	−0.06	−2.65	0.008	−0.28 (0.16)	0.75 (0.55–1.03)	0.075	0.098	1.00	21.66^***^
**Peer relationship problems score**																								
Unadjusted	−0.51 (0.09)	−0.14	−5.94	<0.001	−0.40 (0.15)	0.67 (0.50–0.90)	0.009	−0.72 (0.11)	−0.13	−6.62	<0.001	−0.54 (0.21)	0.58 (0.39–0.87)	0.008	−0.62 (0.08)	−0.17	−7.32	<0.001	−0.71 (0.16)	0.49 (0.36–0.67)	<0.001	0.018	1.00	21.44^***^
Adjusted^b^^,^^d^	−0.50 (0.09)	−0.13	−5.62	<0.001	−0.45 (0.16)	0.64 (0.46–0.88)	0.006	−0.73 (0.11)	−0.14	−6.71	<0.001	−0.63 (0.22)	0.53 (0.35–0.81)	0.004	−0.60 (0.08)	−0.16	−7.15	<0.001	−0.70 (0.16)	0.50 (0.36–0.68)	<0.001	0.063	1.00	13.49^***^
**Prosocial behavior score**																								
Unadjusted	0.02 (0.10)	0.01	0.23	0.817	0.13 (0.14)	1.14 (0.87–1.50)	0.336	0.27 (0.13)	0.04	2.12	0.034	−0.17 (0.18)	0.84 (0.59–1.21)	0.353	0.35 (0.10)	0.08	3.46	0.001	−0.25 (0.14)	0.78 (0.59–1.03)	0.079	0.006	0.996	7.02^***^
Adjusted^b^^,^^d^	−0.10 (0.10)	−0.02	−0.93	0.354	0.18 (0.15)	1.20 (0.90–1.61)	0.214	0.13 (0.13)	0.02	1.04	0.298	−0.10 (0.19)	0.90 (0.62–1.31)	0.585	0.32 (0.10)	0.07	3.21	0.001	−0.21 (0.15)	0.81 (0.61–1.08)	0.147	0.067	1.00	14.37^***^
**Self-esteem score**																								
Unadjusted	0.05 (0.30)	0.004	0.17	0.863	0.05 (0.17)	1.05 (0.75–1.47)	0.787	−0.58 (0.38)	−0.03	−1.54	0.125	−0.12 (0.23)	0.89 (0.57–1.39)	0.608	−0.29 (0.30)	−0.02	−0.97	0.332	0.02 (0.17)	1.02 (0.73–1.44)	0.892	0.001	0.583	1.54
Adjusted^c^^,^^d^	0.12 (0.31)	0.01	0.37	0.710	0.05 (0.19)	1.05 (0.73–1.51)	0.788	−0.36 (0.38)	−0.02	−0.96	0.335	−0.17 (0.23)	0.84 (0.53–1.33)	0.462	−0.35 (0.29)	−0.03	−1.21	0.226	0.04 (0.18)	1.04 (0.74–1.46)	0.826	0.042	1.00	8.78^***^
**Happiness score**																								
Unadjusted	−0.09 (0.10)	−0.02	−0.88	0.378	0.30 (0.22)	1.35 (0.87–2.07)	0.179	−0.48 (0.13)	−0.07	−3.64	<0.001	0.61 (0.25)	1.83 (1.12–3.01)	0.016	−0.11 (0.10)	−0.03	−1.10	0.273	−0.02 (0.23)	0.98 (0.63–1.53)	0.930	0.004	0.97	4.97^*^
Adjusted^c^^,^^d^	−0.10 (0.11)	−0.02	−0.91	0.361	0.27 (0.23)	1.31 (0.83–2.07)	0.246	−0.51 (0.13)	−0.08	−3.81	<0.001	0.61 (0.26)	1.84 (1.10–3.06)	0.019	−0.16 (0.10)	−0.04	−1.52	0.129	−0.003 (0.23)	0.997 (0.64–1.56)	0.988	0.033	1.00	6.67^***^
**Resilience score**																								
Unadjusted	0.69 (0.25)	0.06	2.72	0.006	−0.26 (0.17)	0.77 (0.55–1.07)	0.123	0.42 (0.32)	0.03	1.31	0.191	−0.16 (0.22)	0.85 (0.56–1.30)	0.454	1.00 (0.25)	0.09	4.01	<0.001	−0.37 (0.17)	0.69 (0.49–0.97)	0.031	0.005	0.986	5.73^***^
Adjusted^c^^,^^d^	0.57 (0.25)	0.05	2.24	0.025	−0.26 (0.19)	0.77 (0.53–1.11)	0.158	0.41 (0.31)	0.03	1.32	0.186	−0.24 (0.23)	0.78 (0.50–1.23)	0.285	0.90 (0.24)	0.08	3.74	<0.001	−0.34 (0.18)	0.71 (0.50–1.01)	0.056	0.096	1.00	22.01^***^

*SE, Standard error; OR, Odds ratio; CI, Confidence interval. Reference, Only child group. Significant significance: p-value < 0.05*.

* *p-value < 0.05*,

** *p-value < 0.01*,

*** *p-value < 0.001*.

a *Values for linear regression*.

b *Normal and Borderline range (ref) vs. Clinical range*.

c *Not low (ref) vs. Low (the score < the 10th percentile)*.

d *Adjusted for child's sex, mother's education, mother's age, household income, respondent's K6, parental history of psychiatric disorders, living with grandparents, and school absences due to illness or trauma*.

As for multivariate logistic regression analysis, using the clinical range of the SDQ and 10th percentile cut-off score of the self-esteem, happiness, and resilience, last-borns, middle-borns, and first-borns had decreased odds ratio (OR) of peer relation problems score after adjusting for other covariates (OR = 0.64, 95% Cl 0.46–0.88; OR = 0.53, 95% CI 0.35–0.81; OR = 0.50, 95% CI 0.36–0.68, respectively). After the adjustment, last-borns were still associated with lower total difficulties score (OR = 0.67, 95% Cl 0.50–0.86). Further, middle-borns were significantly associated with unhappiness (OR = 1.84, 95% Cl 1.10–3.09). There was no association between the self-esteem score and birth order.

Comparing the results of the two models, the directions of the association between birth order and each mental health variable in the linear regression model were mostly consistent with the logistic regression model (Normal and Borderline range vs. Clinical range in the SDQ; Normal vs. Low self-esteem, Low resilience, Unhappiness). When compared the linear regression with logistic regression models by birth order, first-borns showed a significant increase in the risk of peer relationship problems in both models and total difficulties in only the linear regression model. Middle-borns showed significant results of a decreased risk of total difficulties, emotional symptoms, and peer relationship problems and an increased risk of unhappiness in both models. Further, last-borns showed a significant decrease in the risk of total difficulties in both models. No statistical significance in hyperactivity/inattention, prosocial behavior, and resilience among last-borns was found in the logistic regression model.

## Discussion

In this study, we found the association between birth order and mental health among children aged 9–10 years. Our results showed that last-borns were less likely to have mental health problems and more likely to have prosocial behaviors and resilience. Middle-borns were found to show the lowest level of happiness, and first-borns were associated with conduct problems. To the best of our knowledge, this study is the first to examine the association of birth order with mental health among pre-adolescent children on both positive and negative sides.

Our findings of last-borns having a lower risk of mental health problems are partially in line with previous research in the UK (47). They identified that later-born children were less likely to have mental health problems than children with no older siblings. Our study focused on the definite birth order position, whereas the UK study assessed the number of siblings, regardless of the order. These findings suggest that the presence of older siblings may play a role in mitigating the risk of mental health problems among children; in turn, last-borns may be in the most advantageous position to receive the benefits by nature. There are several possible explanations for the association. First, interactions with older siblings provide contexts to develop social and emotional competencies, which are known as protective factors for mental health and peer relationship problems (62–64). In daily life, children with siblings usually spend more time with siblings than parents (65). Interactions with siblings promote understanding of others' emotions, thoughts, and intentions (66), which foster their development of social competence (67). Besides, plays and conflicts with siblings develop emotional regulation (68, 69) and problem-solving skills (70, 71). Interactions through teaching, sharing, and cooperation facilitate prosocial behavior (72–74). Given the interactive opportunities, last-borns may have more chances to develop social and emotional competencies from early childhood than other siblings and only one child.

Second, older siblings may play a complementary role in caregiving. When parents are unavailable, siblings can become candidates for attachment relationships and also take on the role of a secure base for younger siblings (75). These relationships provide a sense of security and comfort to younger siblings in insecure family situations (76, 77). Furthermore, last-borns may be more likely to receive more emotional support from older siblings (78). Thus, older siblings can be a source of security for last-borns, which may contribute to the prevention of mental health problems.

Third, parental differential treatment may affect the association of birth order with mental health problems. Parental differential treatment, such as favoritism and unfavorable comparison, can cause not only sibling jealousy (79) but also behavior problems (80), emotional issues (81), and lower self-esteem (82). In Japan, the i.e., system and the practice of *amae* may strengthen parental differential treatment. It may affect mental health more negatively among older siblings. On the contrary, last-borns may be less likely to be affected, which may lead to a decreased risk of their mental health problems. However, the association between these unique characteristics and mental health problems among Japanese children is still unclear. Hence, further research is needed to confirm the association.

However, previous studies have reported that last-born adolescents and adults have an increased risk of mental illness in the US, the UK, Norway, and Sweden (5–8), which is inconsistent with our findings. There are two possible explanations for the inconsistency. The one is that the positive effects of the last position on mental health in childhood may vary in adolescence and adulthood. In adolescence, children spend more time with peers and less with family and have more opportunities to face stressful events and peer relationship problems (83–85). The benefits of the last position within a family may be attenuated in different life stages. The other one is that sociocultural characteristics in each country may contribute to the inconsistency. Japanese sociocultural characteristics, that is *amae* and i.e., system, might account for the inconsistent findings between Japan and other countries. Further longitudinal studies are needed to reveal the mechanism of the inconsistency, comparing with other countries.

Our study also demonstrated that last-borns showed a higher resilience score. Previous studies have reported that the development of resilience is associated with social competence (86), problem-solving skills (87) and self-regulation skills (88), and familial factors such as support availabilities (89, 90). Given the presence of older siblings, last-borns may be more likely to develop these skills through interactions with the siblings and receive emotional support from the siblings, which may contribute to promoting the development of resilience from early childhood.

Furthermore, our findings showed that first-borns were associated with conduct problems, which is partially consistent with a prior study in the UK reporting older siblings having an increased risk of the problems (47). This association may be related to several possible explanations. First, first-borns are required to adopt to siblinghood after the birth of siblings (91). After subsequent sibling birth, first-borns may experience less parental attention and interactions, described as “dethronement” by Adler's theory (66). The behavior problems of first-borns may be considered as a stress response to the change of the home environment (47). Second, parents might change their child-rearing style between the first-born and the subsequent children, which may induce jealousy toward younger siblings (92). That is, parental differential treatment may cause first-borns to suffer from emotional dysregulation, impulsiveness, and vulnerability to frustration (82, 93). In addition, as discussed previously, Japanese sociocultural characteristics may promote the differential treatment. Third, parents may require the first-borns to be a role model for younger siblings. Notably, in Japan, parents tend to expect first-borns to be the heir (32). Consequently, the first-borns may feel more responsibilities and emotional pressure (94). Hence, first-borns may be more likely to feel more emotional distress than younger siblings and only one child and, in turn, have an increased risk of conduct problems.

Moreover, we found that middle-borns showed the lowest level of happiness, compared to only children. Prior research demonstrated that parents rated that last-borns and only children aged 3–9 years were happier than first-borns and middle-borns in the US (20). Our study is the first to report that middle-born children rated themselves as the most unhappy. Kidwell suggested that the middle-borns have a lack of uniqueness in the family and no inherent reasons to receive parental attention and recognition, compared to first-and last-borns (19). In addition, a prior study has demonstrated that middle-born youth tend to perceive less closeness to parents (95) and express less positive attitudes toward family than first-borns and last-borns and more positive views toward friends (96). These findings suggest that the presence of both older and younger siblings may induce perceived unhappiness among middle-borns, and the state of unhappiness in childhood may affect their values toward family and peers in adolescence and adulthood. However, the effects of unhappiness on later life among middle-borns remained unclear; further longitudinal research is needed to examine the trajectories.

Our study found no differential association of birth order with self-esteem among pre-adolescent children. Previous research has shown that first-borns and only children tend to have higher self-esteem than later-borns (5, 97). Further, another study has reported that middle-borns have significantly lower self-esteem than first-borns and last-borns (19). However, it is noteworthy that these studies were conducted more than three decades ago and focused on adolescents and young adults, which may lead to inconsistency with our findings. Self-esteem is found to decline across adolescence through the experience of stressful life events (98), for example, school transitions (84, 85). In Japan, school transitions generally occur between the ages of 12 and 15 years. For this reason, the participants in our study typically have no experience of school transitions. Thus, the experience of other stressful life events may contribute to the inconsistency. Moreover, Baldwin and Hoffmann indicated that perceived family support has preventive effects on the decline of self-esteem in adolescence (99). Given the negative views toward family among middle-borns (95, 96), they may have lower stability of self-esteem when facing stressful events; in turn, they may have an increased risk of a decline in self-esteem in adolescence, compared to other siblings and only children. Further longitudinal research is required to reveal the association between birth order and self-esteem from childhood to adulthood.

This study has several limitations. First, this study did not evaluate the quality of sibling relationships. Poor sibling relationships are found to affect children's mental health negatively (100). Second, we did not differentiate the sibling compositions, such as boy-boy, boy-girl, girl-boy, girl-girl (four types by sex of two siblings), boy-boy-boy, boy-boy-girl, boy-girl-boy, boy-girl-girl, girl-boy-boy, girl-boy-girl, girl-girl-boy, girl-girl-girl (eight types by sex of three siblings), or age difference, say, 1 year difference or 5 years difference, due to limited sample size. Sibling relationships may vary depending on the compositions (92). Sibling compositions might change the magnitude of the effects of birth order on mental health. Third, we could not assess the influence of peer relationships. Peer relationships, such as bullying and victimization, are important factors that affect mental health among school children, and such exposure might vary by sibling positions (101). Thus, the quality of peer relationships might modify the effects of birth order. Fourth, we did not assess families who had experienced perinatal loss through miscarriage, stillbirth, and neonatal death. Prior research reports that the experience influences subsequent pregnancy (102, 103) and parenting behavior (103, 104). Thus, the experience may affect the number of children and parenting styles, which suggests that the effects of birth order among families having the experience may differ from those who have not. Fifth, children's medical issues, such as specific psychiatric disorders and environmental stress, and trauma experience, were not assessed. Also, in this study, children's mental health conditions were not evaluated by more objective measurement tools, such as DSM-5 criteria and the teacher-rated SDQ. Sixth, we did not assess parental personality and parenting styles. These may affect the association between birth order and children's mental health. Finally, this study used cross-sectional data and could not consider past mental health conditions. Further studies are needed to elucidate the effects of birth order on child mental health trajectories, adjusting for parenting style and sibling relationships in a longitudinal design.

Despite these limitations, our findings can help parents identify children at risk for mental health problems in pre-adolescence. Information and guidance about the association of birth order with mental health may allow parents to notice initial symptoms of child mental health problems and manage these problems before adolescence at an earlier stage. In addition, supporting parental rearing practice may also help minimize the adverse effects of birth order on mental health among children. Further, based on birth-order, intervention focusing on both parent-child and sibling relationships may be beneficial to improving mental health among children.

In conclusion, our study found that birth order had differential associations with mental health in both positive and negative aspects among Japanese children aged 9–10. These findings may be helpful to prevent mental health problems depending on birth order in adolescence. Future longitudinal studies are needed to elucidate the mechanisms of the effects of birth order on mental health and the trajectories across the life stages.

## Data Availability Statement

The original contributions presented in the study are included in the article/supplementary material, further inquiries can be directed to the corresponding author/s.

## Ethics Statement

The studies involving human participants were reviewed and approved by Tokyo Medical and Dental University Ethics Committee. Written informed consent to participate in this study was provided by the participants' legal guardian/next of kin.

## Author Contributions

YF contributed to conception, design, analysis, interpretation, drafted, and critically revised the manuscript. SD and AI contributed to conception, design, data acquisition, analysis, interpretation, and critically revised the manuscript. MO contributed to conception, design, data acquisition, and critically revised the manuscript. TF contributed to conception, design, data acquisition, analysis, interpretation, drafted, and critically revised the manuscript. All authors gave final approval and agree to be accountable for all aspects of the work.

## Conflict of Interest

The authors declare that the research was conducted in the absence of any commercial or financial relationships that could be construed as a potential conflict of interest.
